# A Simple Spatial Harmonization Technique by Aggregation for Ecological Studies: An Application Using Swiss Healthcare Regions

**DOI:** 10.1029/2026GH001875

**Published:** 2026-11-06

**Authors:** Jacopo Vanoli, Ana Maria Vicedo‐Cabrera

**Affiliations:** ^1^ Institute of Social and Preventive Medicine University of Bern Bern Switzerland; ^2^ Oeschger Centre for Climate Change Research University of Bern Bern Switzerland

## Abstract

Geographical units are fundamental to epidemiological research, yet their boundaries frequently change over time, creating challenges for long‐term analyses. We propose a simple aggregation‐based framework to harmonize regions with temporally varying boundaries by constructing stable spatial units. The method represents geographic regions from different administrative versions as nodes in a graph and links regions that share underlying postal codes. Connected components of this graph define unified, time‐consistent regions that can be used for downstream analyses without disaggregating outcome data. We demonstrate the approach using the Swiss MedStat Regions (SMR), whose boundaries were substantially revised in 2008, resulting in a many‐to‐many correspondence between old and new definitions. Postal codes, assumed to be largely stable, serve as the bridging key. The resulting harmonized regions (mSMR) were applied to a nationwide time‐series study of mean daily summer temperature and emergency hospital admissions for respiratory diseases among older adults (≥65 years) in Switzerland from 1998 to 2023. The proposed approach is most appropriate when events are sparse at fine scale (e.g., daily counts), exposure can be aggregated to harmonized units, and some loss of spatial resolution is acceptable in exchange for temporal consistency and avoidance of interpolation. Limitations include potential masking of local heterogeneity and susceptibility to the modifiable areal unit problem, particularly for exposures with strong small‐scale spatial gradients. This graph‐based harmonization offers a practical solution for maintaining spatiotemporal consistency in longitudinal epidemiological studies where only aggregate health data are available.

## Plain Language Summary

Public health studies often use maps of administrative regions, such as health districts or postal areas, to analyze how environmental factors like heat affect health. A common problem is that these regions change over time: boundaries are merged, split, or redefined. When this happens, it becomes difficult to compare health risks across long time periods, because the areas used at the beginning of the study no longer match those at the end. In this work, we present a simple and practical method to create regions that remain consistent over time using a network theory approach. As an example, we apply the method to different versions of the Swiss MedStat Regions, combining old and new boundaries into a stable set of regions. We then use these new regions to examine how summer temperatures are linked to hospital admissions for respiratory diseases among older adults in Switzerland.

## Key Points

- We propose an aggregation method to harmonize administrative regions across boundary changes, producing temporally consistent units
- Uses Swiss MedStat Regions as case study, leveraging postcode crosswalks to define connected components as stable analysis units
- Highlights when aggregation is preferable to disaggregation or interpolation and discusses limitations

## 1 Introduction

Geographical units such as administrative regions are an essential part of epidemiological research (Morrison et al., 2024). They provide the spatial framework for understanding how contextual factors shape health outcomes and for examining disease patterns, thereby enabling policymakers to optimally target interventions. The spatial scale of the chosen unit directly influences study results: for example, very small‐area units, such as neighbourhoods, may allow relatively precise identification of health inequalities, disease clusters, and localized environmental exposure‐response relationships, whereas larger administrative regions cannot. However, sometimes information at small‐area level is not available and, even if available, the population size is too small to compute meaningful exposure‐outcome associations (Rao & Molina, 2015). Another issue is that areal boundaries attached to administrative definitions are often modified due to administrative decisions (i.e., unification or separation of municipalities, creation of new neighbourhoods). Spatial consistency is relevant in epidemiological contexts for studying temporal trends and exposure–outcome relationships across long periods.

This issue has been successfully tackled in various ways, depending on the study context. On one side there are interpolation approaches attempting to approximate missing spatial point or areal information using that of neighboring observations (Izaguirre, 2025; Langford, 2013; Lin et al., 2011, 2013). These methods are extremely flexible in terms of both input data sources and output information. However, they often have the drawback of requiring a series of choices from the investigator (e.g., of the spatial resolution in output). We refer to for a comprehnsreview of the methods. Alternatively, “consistent areas through time” (CATTs) were developed (Exeter et al., 2005, 2019) by merging different regional definitions across time in Scotland, solely by overlaying of geographies, without interpolating or approximating. The advantage of the CATT approach is that it aggregates regions and does not distort the original boundaries by interpolation or approximation. However, the procedure is not straightforward to generalize as it is tailored around the Scottish context. Finally, hybrid procedures were also developed (Logan et al., 2014).

The use of stable, comparable units across time may support more robust analyses of changes in disease patterns (Norman & Riva, 2012) thereby facilitating the modeling of time‐varying associations between exposures and health outcomes in time‐series analyses(Norman, 2006). Sometimes it is, thus, beneficial to harmonize geographic units across different time periods to maintain the spatiotemporal consistency of the area unit of analysis.

We present a simple aggregation method for administrative boundaries that allows researchers to use account for territorial changes while preserving the advantages of spatially consistent data, empirically optimizing the use of available data and supporting assessments of environmental and social determinants. As case study we focus on an analysis on heat‐related hospitalizations in Switzerland, defined at Swiss Medstat Regions (SMR) (BFS, 2016). These administrative regions provide a standardised system of geographical units for health statistics. They have been previously used to analyze spatial variations in health outcomes, healthcare utilisation, and environmental exposures (Filippini et al., 2019). However, like many territorial systems, SMR boundaries have been subjected to heavy changes due to administrative reorganisations (in 2008) and we are interested in obtaining temperature‐health associations across their entire timeline.

## 2 Methods

### 2.1 Swiss MedStat Regions and Rationale

The Swiss MedStat Regions (SMR) represent a standardized geographical division of Switzerland based on aggregated postal codes (BFS, 2016). Established in 1998, the SMR system was designed to enable the collection and analysis of hospital discharge data for public health research while preserving patient anonymity. The regions were constructed to be socio‐demographically comparable, though their size and shape vary considerably; that is, larger in sparsely populated or mountainous areas and smaller in densely populated urban areas of the Swiss Plateau (Klauss et al., 2005). In 2008, the SMR boundaries were revised to better align with other national territorial classification systems and to reflect evolving urban development, population distribution, and healthcare infrastructure (BFS, 2016).From this point onward in the paper, we will use the notation “pre‐2008” to refer to the SMR version valid in period 1998–2007 and “post‐2008” for the SMR valid in the period 2008 onward.

The pre‐2008 604 regions were redefined as 705 post‐2008 in the updated version (Haynes et al., 2020; Klauss et al., 2005). Because these changes involved both merging and splitting of regions, the correspondence between the two versions is not one‐to‐one. Instead, several old regions correspond to multiple new ones (with different names), and vice versa, creating a many‐to‐many mapping problem that complicates ecological analyses.

To the best of our knowledge, only one previous Swiss epidemiological study addressed this issue by redistributing the number of cases (e.g., hospitalisations) in each SMR to the underlying postcodes based on the population density (Filippini et al., 2019). For example, if within a SMR there are 10 events (for a specific time unit) and the largest postcode (within that SMR) includes 20% of the population, then the postcode will be assigned to 2 events. This is possible only because postcode codes are consistent (except for a very few exceptions discussed later) in time and between SMR changes, and the information on the population number per postcode is available. A more general procedure based on the disaggregation of English administrative boundaries was previously developed (Norman et al., 2003) to study time‐series of mortality rates in the UK.

Disaggregating event counts per postcodes (or other similar unit) is a suitable approach because no loss of information occurs. In fact, if the exposure varies highly spatially, assigning it at postcode‐level might even reduce misclassification bias (Elliott & Wartenberg, 2004). However, the current methods to assess short‐term effects do not work when the number of event counts at SMR level is too small. For instance, time‐series analyses typically use daily counts which mostly consist of very small numbers that cannot be meaningfully disaggregated into postcodes (i.e., non‐integer values) and are not desirable due to lack of stability and interpretation. To address this issue, we present an alternative framework that harmonizes spatially aggregated health data across time using a simple approach using basic graph theory. The method identifies stable, time‐consistent spatial units by combining regions that have changed definitions over time and maybe particularly useful in time‐series settings (i.e., small space‐time units with limited number of events).

### 2.2 Description of the Proposed Framework

Our method shifts from a disaggregation perspective used by to an aggregation perspective. The core idea we present is grouping of SMR definitions from different versions into larger regions consistent in time, which we will call mSMR (modified‐MSR). Thus, the grouping requirement is that each original SMR (before and after 2008) has to map to only one “new” region. This is achieved by representing each SMR, regardless of version, as a node in a graph (Figure 1a) and identifying the so‐called components (i.e., mSMR). Each node in the old SMR is linked to a node in the new SMR whenever they share at least one postcode. Each set of mutually connected SMRs forms a graph component, which can be identified with standard software and treated as a single, temporally consistent spatial unit for analysis (Diestel, 2025; Filippini et al., 2019). As a result, environmental health models can be fitted on harmonized regions without redefining the outcome data.

**Figure 1:**
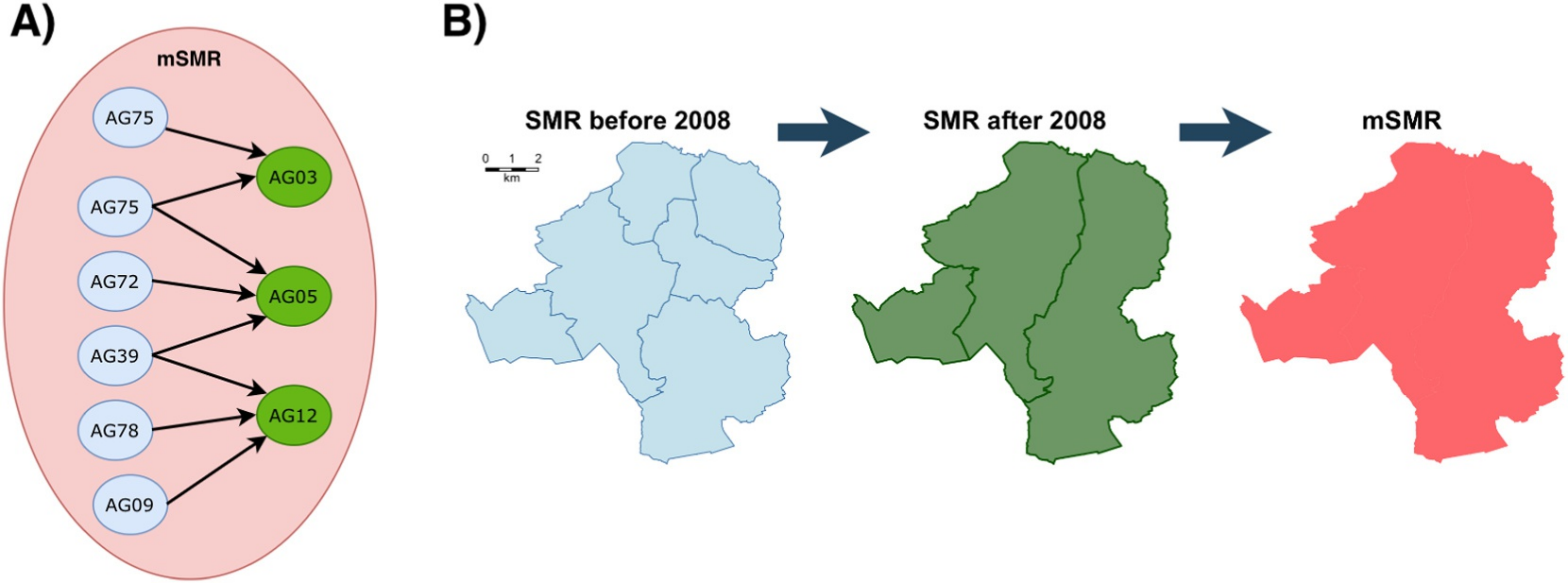
(a) Directed graph of SMR changes: nodes are represented by SMR codes and arrows represent transitions from old (<2008, light blue background) to new (>=2008, green background). This group of connected regions forms a component (i.e., mSMR, red background). (b) Geographical illustration of changes in SMR definitions, showing pre‐2008 regions, post‐2008 regions, and their aggregation into merged regions for consistent epidemiological analyses over time.

### 2.3 Crosswalk Table

To implement the aggregation procedure, a crosswalk table (Wu et al., 2023) is required, sometimes also called conversion table (Simpson, 2002). This table encodes the relationships between pre‐ and post‐2008 SMR definitions, using postal codes as a linking key. Postal codes act as the bridge between SMR versions under two assumptions: (a) postal codes are stable over the study period, so the same code appears in both classifications, and (b) each postal code belongs to only one SMR in each version, with no overlaps. In practice, these assumptions should be checked empirically (e.g., by flagging missing or duplicated postcodes) before constructing the graph. Assumption (a) may not be met for several reasons, such as urban expansion, building demolitions and postal re‐organization (Hutchison, 2026; ONS, 2023). The solution to this issue is highly dependent on the context. In our case study, there are eight instances of incosistencies between postcodes of the two SMR definitions (Table S1 in Supporting Information S1). Due to the low number, we inspected each of them separately. As a result, we observed that all the postcodes involved are small, with population sizes ranging from hundreds to a few thousands people. Moreover, the inconsistencies were attributable to two types of situations: the old postcode (SMR before 2008) was split in two. Otherwise, the postcode only changed its name. In the first case, the two new postcodes always mapped to the same SMR, so the postcode changes did not affect the mapping at Medstat resolution. In the second case, the postcode only changed names and this does not influence the mapping. We decided to keep all the postcodes. Assumption (b) in our context is always met by definition because SMRs were defined as aggregation of postcodes and they cannot overlap.

Another more subtle issue, is that neighbourhoods may be moved from one postcode to another. In general, these changes should be accounted for by using interpolation or other methods (Comber & Zeng, 2019; Logan et al., 2021). However, only postcodes located along the SMR border would affect exposure and outcome information. In this context these changes are unlikely to be spatially very relevant because the unit of aggregation for both exposure and outcome is the SMR, spanning dozens to hundreds of kilometers (Table 1).

**Table 1:** Illustrative Example of Crosswalk Table Corresponding to the Data in Figure 1

SMR (Before 2008)	Old postcode (Before 2008)	New postcode (from 2008)	SMR (from 2008)
AG39	1234	1234	AG05
AG39	5678	5678	AG12
AG71	9101	9101	AG03
AG71	112	112	AG05
AG72	1488	1488	AG05
AG75	2773	2773	AG03
AG78	840	840	AG12

*Note.* It shows the mapping between SMR codes before and after 2008, using postal codes as the linking key (the postcodes are for illustrative purposes only and do not represent real codes).

Table 1 presents an illustrative crosswalk for the regions shown in Figure 1; the postal codes used are hypothetical and do not correspond to real locations.

### 2.4 Stable Spatial Units as Graph Components (mSMR)

Using the crosswalk table, a graph is constructed in which each SMR is a node, and an edge is drawn between any pair of regions that share at least one postal code. In this graph, regions connected through shared postcodes, regardless of whether they belong to the pre‐ or post‐2008 definitions, form connected components (Figure 1a). The final output of this procedure is a set of harmonized spatial units, one for each connected component. These units serve as the building blocks for all downstream statistical analyses. Exposure data can be aggregated to these stable regions, health outcomes can be aggregated without modification, and standard regression models can be applied.

### 2.5 Association Between Heat and Risk of Respiratory Hospital Admissions

The proposed aggregation method was applied to assess the risk of hospital admissions due to heat across the whole timeline of the Swiss Medstat regions. Therefore, we conducted a time‐series analysis and compared the estimated exposure–response (ER) associations using different spatial definitions (i.e., pre/post change with and without aggregation). Because this work mainly focuses on the harmonization method, we limited the scope of our epidemiological analysis by selecting a study sample and a specific hospitalization cause with prior evidence of positive ER associations (Kenny et al., 2010; Lee & Vicedo‐Cabrera, 2026; Ragettli et al., 2024) and high statistical power. Specifically, we examined the short‐term association between mean daily summer temperature (May–September) on emergency hospital admissions for respiratory causes (ICD‐10: J1–J99) among older adults (≥65 years) in Switzerland in the period 1998–2023. To assess the robustness of the method in local areas, we also run an analysis restricted to a single Swiss canton. The Canton of Zurich was chosen due to its comparatively large sample size relative to the other cantons. We constructed exposure and outcome data sets using multiple definitions of the SMR. Two original definitions were considered: “pre‐2008,” applied to the period 1998–2007, and “post‐2008,” applied to 2008–2023 (the latest hospitalization data available). In the main analysis, the newly defined regions “mSMR” were applied to the entire follow‐up period (1998–2023). Daily mean temperatures were obtained from MeteoSwiss (MeteoSwiss, 2026). For the exposure, we computed unweighted and population‐weighted daily averages, each analyzed in separate analyses. We collected population data from NASA Gridded Population of the World data set (NASA GPWv3, 2025) at 1 × 1 km resolution. Temperature was averaged, and hospitalization counts were summed to the SMR level. More details on the population data and weighting can be found in Text S1 of Supporting Information S1.

We applied a time‐series design using a quasi‐Poisson distributed lag non‐linear model framework for small‐area analysis (Gasparrini, 2022). Specifically, we selected the regression model specifications and covariates to ensure control for potential temporal confounding, based on similar epidemiological studies in the existing literature (Gasparrini et al., 2022; Ragettli et al., 2019). We stratified the analysis by SMR, year and month:

g[E(y)]=ξ+f(temperature,lag;θ)+s(doy;γ)∗year+I(w;ϕ).

Where the *g* represents the logarithm link function, *E*(*y*) the outcome expected value. The letter *ξ* represents the intercept, *s*(doy; ***γ***)*year a 4 degrees of freedom natural spline‐by‐year interaction and *I*(*w*; ***ϕ***) an indicator for day of the week. This was done to adjust both for seasonal patterns and yearly changes in the temperature distribution. Finally, the exposure‐lag association with 7 lags was modeled with a cross‐basis *f*(*x*, lag; ***θ***) defined by natural cubic spline with internal knots at the 50th and 90th percentiles of the exposure distribution, while the lag–response dimension used a natural spline with 2 internal knots. The letters ***γ***, ***ϕ***, and ***θ*** represent the estimated coefficients for each predictor specification (e.g., natural spline). The results were expressed in terms of relative risk estimates (RR) with 95% confidence intervals across the observed exposure range, centered at 20°C, considered approximately the optimal temperature (Ragettli et al., 2024).

We acknowledge the use of ChatGPT, Claude, and Perplexity AI for assistance with grammar and syntax correction, code development and debugging, and figure creation. All resulting content was reviewed and validated by the authors.

## 3 Results

The descriptive table (Table 2) of the SMR characteristics shows that the number of regions substantially increased with the 2008 revision (from 604 pre‐2008 to 705 post‐2008). However, this increase did not translate into a marked reduction in the average size of the spatial units, as the mean area remained around 50–60 km^2^ and the median area increased from 18.1 to 23.3 km^2^, indicating a heterogeneous redistribution of area sizes rather than uniform spatial refinement. In contrast, the merged SMR (mSMR) configuration collapsed pre‐ and post‐2008 postcodes into 303 larger regions, more than doubling the mean area (132.2 km^2^) and increasing the median area to 28.0 km^2^, while encompassing all 544,339 hospitalizations observed over the study period. The changes are also detectable on the maps (Figure 2) where we can observe that the aggregation in some cases leads to extremely large regions.

**Table 2:** Basic Descriptives of the SMR Regions by Version, Including Prior to 2008 (Pre‐2008) Changes, After 2008 Changes (Post‐2008) and After the Proposed Aggregation Procedure (mSMR)

SMR version	n. of regions	Mean area (km^2^)	Median area (km^2^)	n. of hospitalizations
Pre‐2008	604	56.54	18.06	125,484
Post‐2008	705	51.68	23.31	418,855
mSMR	303	132.24	28.00	544,339

**Figure 2:**
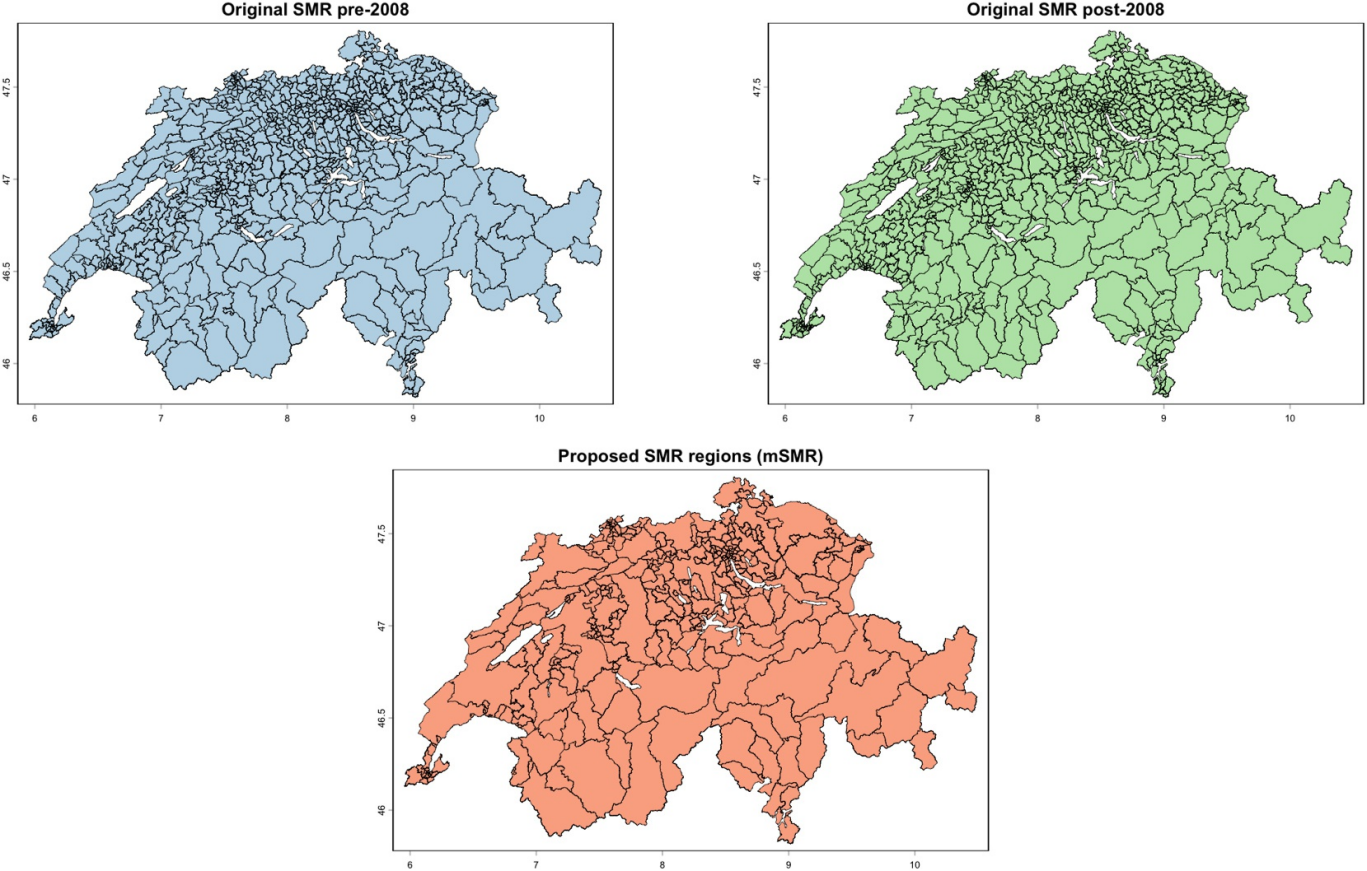
Maps of the three Swiss Medstat Regions (SMR) versions. In light blue, the regions pre‐2008 changes. In green, the regions post‐2008 changes and in orange the regions after applying the proposed harmonization method (mSMR).

Between 1998 and 2023, a total of 544,339 hospitalizations were recorded. Of these, 125,484 occurred before 2008, representing approximately 23% of all admissions. Figure S3 in Supporting Information S1 displays the distribution of temperature by period and SMR definition, showing a strong overlapping among all definitions. Figure 3 displays the estimated exposure‐response (*E*‐*R*) functions for the two original SMR definitions (pre‐2008 and post‐2008) and for the newly defined regions. The association appears stronger before 2008 (red curve). The association estimated for the new regions (blue curve) closely resembles the average of the two original definitions. This pattern empirically suggests that defining the exposure effect in the merged regions as the average of the period‐specific associations may appropriately capture temporal variability across the follow‐up. Additional analyses comparing the associations estimated in the new regions with those obtained using the original MSRs in their corresponding follow‐up periods show that the effect estimates remain essentially unchanged when applying the new regional definition, both at national (Figure 4) and Zurich canton level (Figure S2 in Supporting Information S1). Furthermore, sensitivity analyses (Figure S1 in Supporting Information S1) indicate no meaningful difference in epidemiological estimates when using population‐weighted versus unweighted temperature averages.

**Figure 3:**
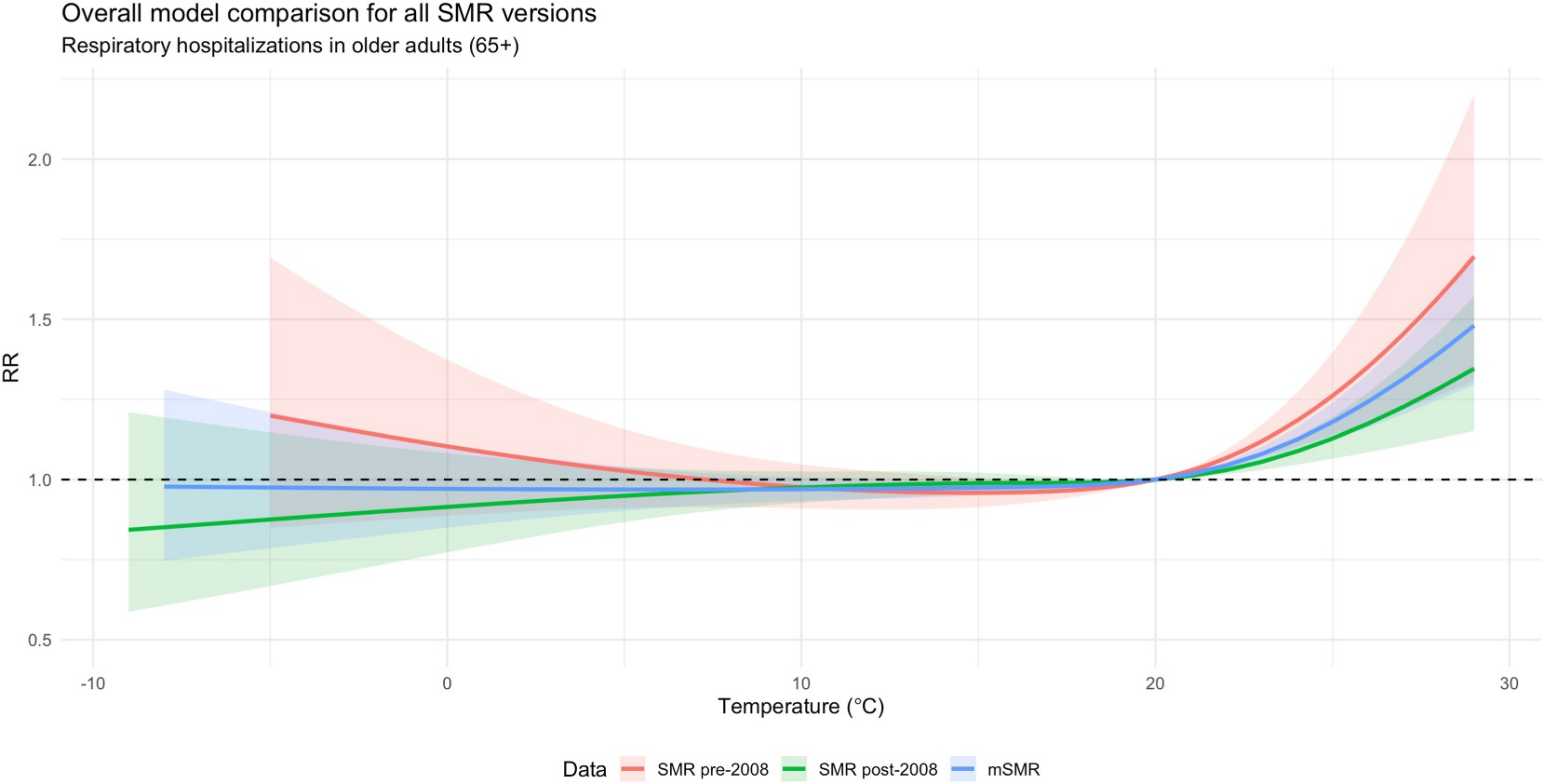
Comparison of temperature‐respiratory hospitalization associations across three spatial definitions and temporal periods. The curves show relative risks (RR) for older adults (65+) associated with daily population‐weighted temperature using Medstat regions pre‐2008 (red, for period 1998–2007), Medstat regions post‐2008 onward (green, for period 2008–2023), and newly defined regions for entire follow‐up (blue, for period 1998–2023). Solid lines represent estimated temperature‐RR relationships, while shaded areas indicate 95% confidence intervals. The horizontal dashed line marks the null value (RR = 1), corresponding to no association.

**Figure 4:**
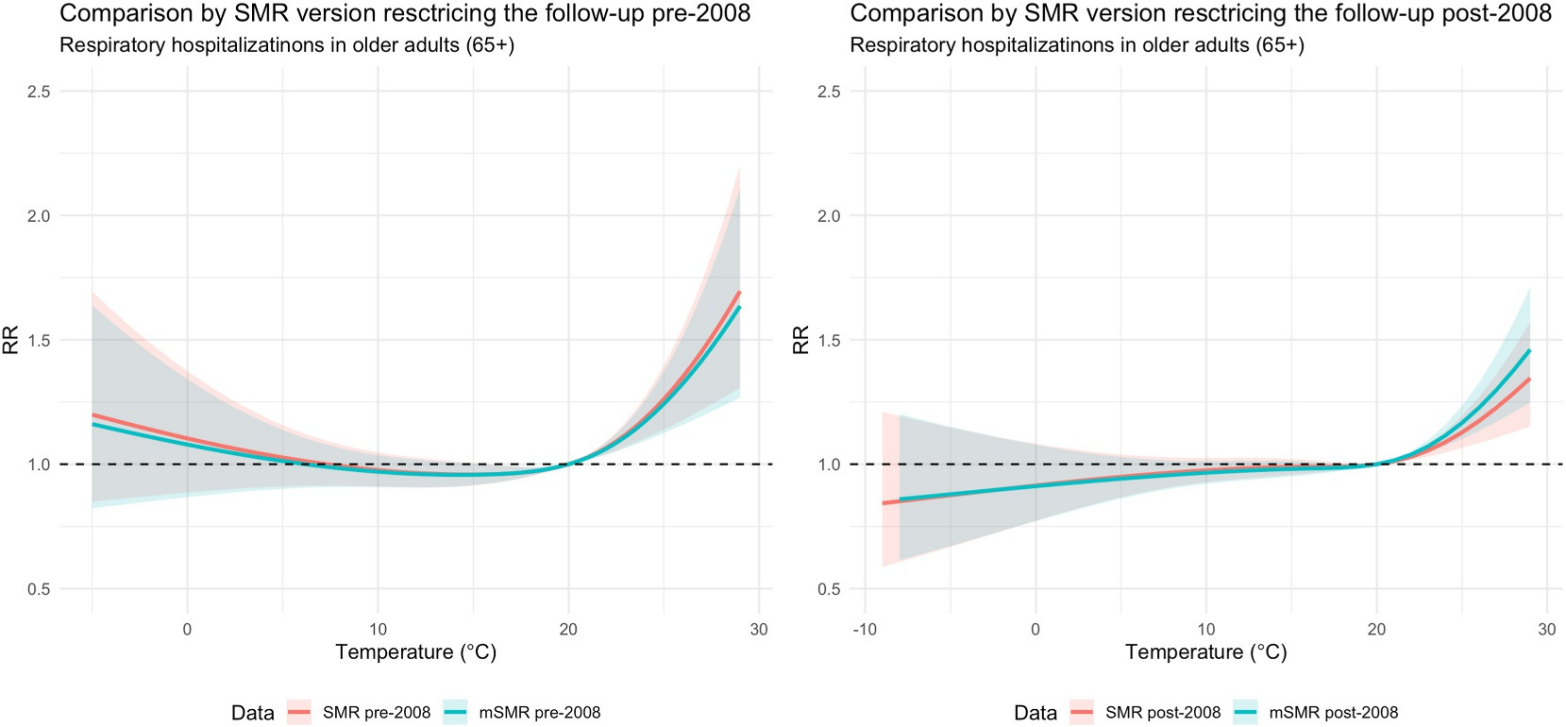
Comparison of temperature–respiratory hospitalization associations in older adults (65+) using different regional definitions before and after 2008. The left panel shows associations estimated using SMR pre‐2008 (red) and the corresponding new region definitions (blue) in the period 1998–2008. The right panel shows estimates for SMR regions from 2008 onward (red) compared with mSMR definitions after 2008 (blue). Curves represent the estimated relative risks (RR) across the temperature range, and shaded areas indicate 95% confidence intervals. The dashed horizontal line marks the null value (RR = 1), corresponding to no association.

## 4 Discussion

The objective of this work was to present a simple and practical approach for integrating routine healthcare area‐level data collected under different administrative versions of geographic regions. The proposed method merges old and new connected regions into unified areas using the basic graph‐theoretical concept of components. As motivational case study, we applied this approach to the Swiss MedStat Regions (SMR) system, created in 1998 and substantially revised in 2008 (BFS, 2016). By harmonizing pre‐ and post‐2008 data within a modified‐SMR framework (mSMR), our approach aims to enable epidemiological analyses across the entire follow‐up (1998–2023) period without the need to stratify by boundary definitions or restrict to specific subperiods.

We used the mSMR in the Swiss context under standard time‐series epidemiological analyses. The resulting exposure–response (*E*‐*R*) functions showed that analyses based on the mSMR closely approximate the *E*‐*R* estimates we would obtain by averaging *E*‐*R* estimates from analyses performed on data before and after 2008. Moreover, if we restrict the analysis to each subperiod, mSMR‐based estimates are highly consistent with estimates using the original SMR definitions both a national and cantonal level. These findings support the use of the SMR regions in temperature‐hospitalizations context, and also suggest that the temperature–mortality association is not markedly affected by the aggregation of data into larger areas.

This method offers several advantages. It is suitable for epidemiological assessments of short‐term and long‐term effects of environmental hazards or other risk factors that change over time. The framework can also facilitate consistent analyses of time‐varying relationships, a relevant aspect in recent heat–health research for assessing adaptation (Achebak et al., 2023; Arbuthnott et al., 2016). More generally, it can be used to evaluate the sensitivity of exposure–outcome associations to changes in temporal or even spatial resolution.

On the other hand, successful reproducibility of this method depends on the nature of the study. In fact, our promising epidemiological results are plausible in this context, where neighboring SMR regions share similar temperature distributions and sociodemographic characteristics (Ragettli et al., 2023). However, this assumption may not hold for different exposures, such as air pollution, and in general in other settings with high spatial heterogeneity in exposure, outcomes, or confounders. In fact, regional aggregation could mask important local variations in associations, reflecting the modifiable areal unit problem (MUAP, Buzzelli, 2020). This issue occurs systematically when aggregation methods are applied and no standard solution exists. However, its relevance depends on the study research question. In our study, we were not interested in disentangling local effects, but rather to provide an overall view of heat‐hospitalizations risk across Switzerland since the SMR were created, possibly at cantonal level. Naturally, if the question aims to investigate the different gradients of effect within each canton (at smaller administrative unit), for instance to identify areas of major vulnerability, this method would not be an appropriate choice. The severity of the MUAP depends also on the characteristics of the exposure in terms of spatial variability. For example, if we focused on studying the short‐term effects of NO_2_ on hospilization risk, it would have been preferable to avoid aggregating regions, because NO_2_ concentrations are highly spatially varying (Hoek et al., 2008). Finally, if the MUAP is suspected, a potentially useful approach to confirm the hypothesis would be removing a random set of the original units (e.g., SMRs), aggregate the remaining ones and estimate the association of interest. This process would have to be reiterated several times to see how the associations are sensitive to the random exclusion of some units (Buzzelli, 2020). Recently, on the same line, additional frameworks have been proposed involving heavy use of simulations (Nieves et al., 2024; Tuson et al., 2019).

Our approach shares several similarities with the Scottish CATTs (Exeter et al., 2005, 2019). In fact, both approaches favor a merging strategy whereby if a source zone overlaps more than one target zone, the affected zones are merged, producing genuinely consistent units. However, they differ substantially in implementation and generalizability. CATTs rely on a GIS‐based polygon overlay of three successive census geographies combined with point‐in‐polygon checks against residential address data to distinguish real boundary changes from “sliver” artifacts, an intensive process that produced three alternative zone configurations (CATT0/1/2) which the authors themselves note is tailored to the Scottish postcode‐based census system and not transferable to, for example, England and Wales. Our mSMR approach achieves a similar outcome but through a simpler graph‐theoretic perspective: rather than overlaying digitised boundaries and manually resolving slivers via address‐point data, we represent each SMR version as a node and link nodes sharing at least one postcode, with connected components directly defining the harmonized units. This avoids the need for detailed boundary geometries, sliver‐elimination heuristics, or multiple candidate configurations, and depends only on a postcode crosswalk table, which is more widely available across administrative contexts than the polygon and address‐point data sets CATTs require. The trade‐off is that, like CATT0, our method offers no control over the resulting unit sizes, and aggregation driven by many‐to‐many postcode links can in principle produce very large or unevenly sized mSMRs (as we also observe in Figure 2).

Finally, our method is also comparable with spatial interpolation approaches as they both share the goal of reconciling mismatched spatial unit, but they operate differently. Whereas the interpolation methods (Comber & Zeng, 2019) estimate values at a different (often finer) spatial resolution than the source data, typically requiring auxiliary information (e.g., population or land cover) and a series of subjective modeling choices, our aggregation‐based mSMR framework avoids estimation altogether by grouping original administrative units into time‐consistent components using a graph‐theoretic approach based purely on shared postal codes. No values are predicted or redistributed, and no ancillary covariates are needed, so the original boundaries are preserved without distortion. In this sense, the two approaches are complementary: interpolation techniques are best suited when finer spatial resolution or a fixed reporting geography is required despite zone mismatches, while our aggregation approach is preferable when the goal is a stable, administratively consistent unit for longitudinal analysis and the loss of some spatial resolution (through merging into larger mSMR units) is an acceptable trade‐off for avoiding the assumptions and approximation error inherent in interpolation‐based redistribution.

As noted in the previous sections, our approach assumes relative spatial and temporal stability of both postcodes names and areas, assumptions implicit in most area‐based epidemiological designs. Minor violations (few changes) of either assumption are unlikely to introduce substantial bias in studies where the spatial unit of interest is considerably larger (several dozens kilometers) than a neighborhood and can be mitigated by manually addressing each change or excluding affected postcodes. However, if the changes in the postcodes names involve a relevant portion of postcodes, the reason for that should be inspected and it may require the use of more advanced harmonization methods mentioned above (Comber & Zeng, 2019).

Moreover, statistical analyses conducted at higher administrative levels may require careful alignment if regional boundaries evolve differently across hierarchical units. For instance, in Switzerland, Cantons, aggregates of SMRs, are often the administrative level at which policy decisions are made. When deriving Canton‐level epidemiological measures, it is important to ensure that SMRs do not shift between Cantons, as such realignments would lead to inconsistent regions. In practice, these cases are rare and can be handled by excluding the few affected regions. This situations are likely more frequent in developing countries due to the rapid pace of urban expansion, the growth and formalization of informal settlements (Samper et al., 2024), and ongoing administrative reorganizations of local government units (Ncube & Monnakgotla, 2016).

Finally, we identified a analogous approach to our method implemented in a different context (Sarkar et al., 2024). However, we emphasize that our approach was developed independently and provides a formal framework, explicit assumptions, and a detailed epidemiological application.

To summarize this section, we provide the following recommendations for when we believe our aggregation procedure is appropriate:

- Outcome data are event counts (e.g., daily hospitalizations) that are too small to be meaningfully disaggregated to finer units (e.g., postcodes).
- Exposure data (e.g., environmental/climate variables) can be aggregated to the same harmonized units without requiring redefinition of the outcome data, enabling standard regression/time‐series models to be applied directly.
- The research question involves a longitudinal analysis or time‐series and would benefit from a single, temporally consistent spatial unit, rather than separate analyses per period.
- Some loss of spatial resolution (administrative regions becoming larger after merging) is an acceptable trade‐off for gaining temporal consistency and analytical stability.
- A finer‐unit crosswalk table linking the old and new administrative definitions is available, and the units are reasonably stable over time (as in the SMR case).
- Preserving the original boundaries without statistical estimation/interpolation is preferred. That is, when avoiding the assumptions, ancillary data requirements, and approximation error of interpolation‐based methods is a priority.
- The many‐to‐many mapping between old and new units is relatively limited in scope (few changed regions), so that the resulting merged units remain reasonably sized.

## 5 Conclusions

We thoroughly described and discussed an established harmonization procedure of geographical units that change in time. We proposed an implementation using a graph‐based strategy. Therefore, we compared it with other spatial harmonization procedures mainly focusing on the best contexts of use. The approach presented provides a practical and effective solution to enable spatially consistent statistical analysis across time, which can be applied to exposure‐outcome epidemiological studies. The method is particularly suited when the basic unit underlying the changes remain stable in time, while other more sophisticated methods are more suitable when the geographical configuration has frequent changes. Overall, we observe that harmonization methods cannot be transferred blindly across different administrative contexts; they consistently require context‐specific adaptation to remain valid and effective.

## Conflict of Interest

The authors declare no conflicts of interest relevant to this study.

## Supporting information

Supporting Information S1

## Data Availability

R Code and geospatial data to reproduce the harmonization procedure are freely available at (Vanoli, 2026) linked to the Github repository https://github.com/JacopoVanoli/medstatlink. For the epidemiological analyses, the hospitalization data is restricted and must be requested to the Federal Statistical Office at info@bfs.admin.ch. The temperature are free to access under request to MeteoSwiss.
