# Supplementary material for: A Simple Spatial Harmonization Technique by Aggregation for Ecological Studies: An Application Using Swiss Healthcare Regions
Source: Geohealth. 2026 Nov 6;10(11):e2026GH001875. doi: 10.1029/2026GH001875 (PMC13741205; doi:10.1029/2026GH001875)
Supplement: Supplementary file 1 [file GH2-10-e2026GH001875-s001.docx]

*GeoHealth*

Supporting Information for

**A simple spatial harmonization technique of aggregated data for ecological studies**

Jacopo Vanoli^1,2^, Ana Maria Vicedo-Cabrera^1,2^

^1^Institute of Social and Preventive Medicine, University of Bern, Switzerland, ^2^Oeschger Centre for Climate Change Research, University of Bern , Switzerland

**Contents of this file**

Text S1

Table S1

Figures S1 to S3

**Introduction**

The support information includes a short paragraph and four figures.

- Text S1 provides details about the exposure processing
- Table S1 provides details about inconsistency in postcodes between SMR versions.
- Figures S1 and S2 display exposure-response estimates
- Figures S3 displays the temperature distribution

**Text S1: Weighting temperature for population density**

SMR regions can span several hundred square kilometers and may exhibit substantial heterogeneity in population distribution. As a result, unweighted averaging of 1 × 1 km gridded temperature estimates across these areas may not accurately represent population-level exposure. To address this limitation, we calculated population-weighted average temperatures, a commonly used approach in environmental epidemiology. Grid-cell–specific temperature values were weighted by the corresponding population density, such that areas with higher population contributed more to the regional mean.

The population data used for the weighting were obtained from the NASA Gridded Population of the World (GPW) dataset at 1 × 1 km spatial resolution, available at 5-year intervals. From that, single annual population surfaces were derived by linear interpolation between consecutive GPW datasets. Additional details on data processing and implementation are provided in the accompanying R code. The code is published on Zenodo (Vanoli 2026) linked to the Github repository (<https://github.com/JacopoVanoli/medstatlink>). Within the repository, the main folder includes the scripts to reproduce the harmonization procedure and the epidemiological analysis. The subfolder “processtemp” includes only the scripts for processing the weighted temperature averages used in the statistical analysis.

**Table S1**: List of inconsistent in postcodes between the two SMR versions.

| **SMR**  **From 2008** | **postcodes**  **From 2008** | **postcodes**  **After 2008** | **SMR**  **Before 2008** |
| --- | --- | --- | --- |
| BE11 | 3049 | 3044 | BE17 |
| BE25 | 3238 | 2076 | BE42 |
| GR13 | 7407 | 7417 | GR06 |
| SG11 | 9300 | 9303 | SG01 |
| TG19 | 8582 | 8580 | TG14 |
| VD48 | 1046 | 1414 | VD37 |
| VD54 | 1047 | 1413 | VD41 |
| ZH79 | 8543 | 8544 | ZH48 |

**Supplementary figures**

Figure S1. Comparison of temperature–respiratory hospitalization associations across different SMR versions. Relative risks (RR) for older adults (65+) are shown for both population-unweighted and weighted temperature metrics across five spatial definitions: SMR < 2008, SMR ≥ 2008, mSMR, mSMR < 2008, and mSMR ≥ 2008. Solid lines indicate estimated RR across temperature, and shaded areas represent 95% confidence intervals. A reference value of RR = 1 (horizontal dashed line) indicates no association.

**Figure S2.** Comparison of temperature–respiratory hospitalization associations in older adults (65+) using different SMR versions restricting the follow-up pre- and post-2008 only within the Canton of Zurich. The interpretation of the legend is analogous to Figure 4 in the manuscript.

**Figure S3.** Density plot of the distribution of daily temperature for different SMR definitions.

**References**

Vanoli, Jappo. 2026. JacopoVanoli/Medstatlink: Medstatlink | Zenodo. R software. V. v1. Zenodo, released. https://doi.org/https://doi.org/10.5281/zenodo.22913587.
